# Vitamin B_12_ insufficiency induces cholesterol biosynthesis by limiting s-adenosylmethionine and modulating the methylation of SREBF1 and LDLR genes

**DOI:** 10.1186/s13148-015-0046-8

**Published:** 2015-02-27

**Authors:** Antonysunil Adaikalakoteswari, Sarah Finer, Philip D Voyias, Ciara M McCarthy, Manu Vatish, Jonathan Moore, Melissa Smart-Halajko, Nahla Bawazeer, Nasser M Al-Daghri, Philip G McTernan, Sudhesh Kumar, Graham A Hitman, Ponnusamy Saravanan, Gyanendra Tripathi

**Affiliations:** Division of Metabolic and Vascular Health, Clinical Sciences Research Laboratories, Warwick Medical School, University Hospital Coventry and Warwickshire, University of Warwick, Clifford Bridge Road, Coventry, CV2 2DX UK; Centre for Diabetes, Blizard Institute, Queen Mary University of London, 4 Newark Street, London, E1 2AT UK; Metabolic Research Laboratories, Wellcome Trust-MRC Institute of Metabolic Science, Level 4, University of Cambridge, Addenbrooke’s Hospital, Box 289, Cambridge, CB2 0QQ UK; Nuffield Department of Obstetrics and Gynaecology University of Oxford Level 3, John Radcliffe Hospital, Oxford, University of Oxford, Oxford, OX3 9DU UK; Warwick Systems Biology, University of Warwick, Gibbet Hill Road, Coventry, CV4 7AL UK; Biochemistry Department, College of Science, King Saud University, Riyadh, 11451 Saudi Arabia; iDEA Centre, George Eliot Hospital, Nuneton, CV10 7DJ UK

**Keywords:** Vitamin B_12_, Folic acid, Cholesterol, Homocysteine, Adipocyte, Methylation

## Abstract

**Background:**

The dietary supply of methyl donors such as folate, vitamin B_12_, betaine, methionine, and choline is essential for normal growth, development, and physiological functions through the life course. Both human and animal studies have shown that vitamin B_12_ deficiency is associated with altered lipid profile and play an important role in the prediction of metabolic risk, however, as of yet, no direct mechanism has been investigated to confirm this.

**Results:**

Three independent clinical studies of women (i) non-pregnant at child-bearing age, (ii) in early pregnancy, and (iii) at delivery showed that low vitamin B_12_ status was associated with higher total cholesterol, LDL cholesterol, and cholesterol-to-HDL ratio. These results guided the investigation into the cellular mechanisms of induced cholesterol biosynthesis due to vitamin B_12_ deficiency, using human adipocytes as a model system. Adipocytes cultured in low or no vitamin B_12_ conditions had increased cholesterol and homocysteine levels compared to control. The induction of cholesterol biosynthesis was associated with reduced s-adenosylmethionine (AdoMet)-to-s-adenosylhomocysteine (AdoHcy) ratio, also known as methylation potential (MP). We therefore studied whether reduced MP could lead to hypomethylation of genes involved in the regulation of cholesterol biosynthesis. Genome-wide and targeted DNA methylation analysis identified that the promoter regions of SREBF1 and LDLR, two key regulators of cholesterol biosynthesis, were hypomethylated under vitamin B_12_-deficient conditions, and as a result, their expressions and cholesterol biosynthesis were also significantly increased. This finding was further confirmed by the addition of the methylation inhibitor, 5-aza-2′-deoxycytidine, which resulted in increased SREBF1 and LDLR expressions and cholesterol accumulation in vitamin B_12_-sufficient conditions. Finally, we observed that the expression of SREBF1, LDLR, and cholesterol biosynthesis genes were increased in adipose tissue of vitamin B_12_ deficient mothers compared to control group.

**Conclusions:**

Clinical data suggests that vitamin B_12_ deficiency is an important metabolic risk factor. Regulation of AdoMet-to-AdoHcy levels by vitamin B_12_ could be an important mechanism by which it can influence cholesterol biosynthesis pathway in human adipocytes.

**Electronic supplementary material:**

The online version of this article (doi:10.1186/s13148-015-0046-8) contains supplementary material, which is available to authorized users.

## Background

Adipose tissue (AT) plays a central role in integrating energy metabolism and glucose homeostasis [1]. Besides being the major site of fatty acid storage as triglycerides (TG), AT is also the body’s largest cholesterol pool. In AT, nearly all cholesterol (>95%) exists as free, non-esterified form and resides in the plasma membrane or at the cytosolic interface of lipid droplets [2-4]. In contrast, other specialised cells and tissues store small pools of esterified cholesterol, such as adrenal cells or foam cells, which have the capacity to accumulate considerable quantities of excess cholesterol esters [5]. A delicate balance exists between uptake, synthesis, and storage which tightly controls the abundance of free cholesterol in peripheral cells [6] while excess free cholesterol is in fact deleterious to cells [7]. AT from ob/ob mice exhibits an increase in cholesterol biosynthesis [8], and hypertrophied adipocytes from obesity rodent model, Zucker rats have elevated mRNA expression of SREBP-2, 3-hydroxy-3-methylglutaryl-CoA reductase (HMGCR), and the LDL receptor (LDLR) [9,10]. Studies in Zucker rats [11] and 3T3F442A cells [12] also highlight that with an increase in triglyceride storage, cholesterol is redistributed from the plasma membrane to the surface of the lipid droplet and adipocyte cholesterol levels appear to increase in proportion to the triglyceride content [13]. There is also accumulating evidence that adipose cholesterol imbalance is closely associated with adipocyte dysfunction and obesity-mediated metabolic complications and insulin resistance [9,13,14].

Despite the significant physiological importance of adipocyte cholesterol, especially with regard to the reported association between obesity and mortality from cardiovascular diseases [15], the regulation of *de novo* synthetic pathway of cholesterol within adipocytes has not been studied in detail. Kumar *et al*. [16] demonstrate that in Wistar rats, offspring from vitamin B_12_ (B_12_) deficient mothers had higher adiposity and altered lipid metabolism and hypothesised that this could be due to dysfunctional adipocyte. These offspring had higher total cholesterol, triglycerides, IL-6, and TNF-α and had lower adiponectin and leptin compared with control offspring. In a prospective, longitudinal study of humans in early pregnancy, vitamin B_12_ deficiency independently predicted gestational diabetes and type 2 diabetes (T2D) at 5-year post-delivery. This reported association was mediated through adiposity highlighting the potential role of low vitamin B_12_-induced adipocyte dysfunction [17]. Furthermore, maternal vitamin B_12_ deficiency also predicted insulin resistance in a 6-year-old offspring, suggesting a role in foetal programming [18]. The vitamins folic acid, B_12_, B_6_, and B_2_ participate in one carbon metabolism as source of coenzymes. One carbon metabolism is a network of interrelated biochemical pathways that donate and regenerate one-carbon units, including the methyl group (reviewed [19,20]). Vitamin B_12_ functions as a coenzyme for 5-methyltetrahydrofolic acid (MTHF)-dependent methionine synthase (MS) which catalyses the synthesis of methionine from homocysteine [21,22]. Methionine is then converted to s-adenosylmethionine (AdoMet), a universal methyl group donor for methylation of DNA and RNA [23].

Restriction of methyl donors, including vitamin B_12_ and folate in pregnant sheep increases offspring’s metabolic risk, likely mediated by altered DNA methylation [24]. In metazoans, low levels of methyl donor AdoMet and phosphatidylcholine activate SREBP-1 and induce lipogenesis [25]. Both vitamin B_12_ and folic acid play important roles in regulating the levels of AdoMet and homocysteine [26,27]. In the current study, we investigated the potential clinical relevance on circulating lipid profiles utilising the data from two different cohorts: the British National Diet and National Survey (NDNS), and women subjects from Saudi Arabian population and found significant associations between cholesterol, triglycerides, and vitamin B_12_ deficiency. Therefore, this current study was designed to understand the molecular mechanisms of how vitamin B_12_ restriction may be inducing these effects. We used human adipocytes as the model system and show that vitamin B_12_ restriction induced cholesterol biosynthesis in adipocytes by limiting cellular AdoMet levels and modulating the methylation of genes which regulate cholesterol biosynthesis. This was further confirmed in human adipose tissue obtained from vitamin B_12_ deficient and sufficient women undergoing caesarean section.

## Results

### Association of low vitamin B_12_ and lipid profile in clinical studies

Our interest was to investigate the association between vitamin B_12_ and lipid profile in the women of reproductive age and/or during pregnancy due to the potential relevance to the offspring. We had access to the two cohorts which fulfilled the criteria: (1) NDNS, a publically available dataset from the UK and (2) pregnant women from Saudi Arabia.

#### NDNS

In NDNS survey, 1,256 subjects had full biochemical analysis which included lipid profiles, B_12_, folic acid, and homocysteine. Three hundred fifteen women were of reproductive age (19 to 39 years). The prevalence of serum vitamin B_12_ insufficiency (<148 pmol/L) was 12%. Women with low B_12_ had significantly higher cholesterol, LDL cholesterol, cholesterol-to-HDL ratio, and homocysteine than normal B_12_ women. The BMI was similar in both groups (Table 1).

**Table 1:** **Clinical characteristics of child bearing group (female 19 to 39 years) from NDNS and pregnant women from Saudi population**

	**NDNS**			**Saudi cohort**		
	**Total**	**B**_**12**_ **(ng/L)**		**Total**	**Maternal B**_**12**_ **(ng/L)**	
	***n*** **= 315**	**>190 (** ***n*** **= 278)**	**≤190 (** ***n*** **= 37)**	***n*** **= 152**	**>190 (** ***n*** **= 84)**	**≤190 (** ***n*** **= 68)**
Age (years)	31.2 ± 0.32	31.7 ± 0.34	30.5 ± 0.94	28.6 ± 0.43	28.5 ± 0.59	28.5 ± 0.62
BMI (kg/m^2^)	25.3 ± 0.29	25.2 ± 0.30	26.3 ± 1.0	26.75 ± 0.43	26.0 ± 0.55	27.8 ± 0.66*
WHR	0.77 ± 0.003	0.77 ± 0.003	0.79 ± 0.01	-	-	-
Total-C (mmol/L)	4.84 ± 0.06	4.79 ± 0.06	5.20 ± 0.21*	5.98 ± 0.09	5.80 ± 0.12	6.20 ± 0.15*
LDL-C (mmol/L)	3.58 ± 0.06	3.52 ± 0.06	4.01 ± 0.21**	3.02 ± 0.14	2.99 ± 0.18	3.14 ± 0.21
HDL-C (mmol/L)	1.26 ± 0.02	1.27 ± 0.02	1.19 ± 0.06	1.25 ± 0.03	1.25 ± 0.04	1.24 ± 0.04
Total-C:HDL ratio	4.11 ± 0.08	4.03 ± 0.08	4.70 ± 0.26**	5.11 ± 0.14	4.99 ± 0.18	5.29 ± 0.22
TG (mmol/L)	-	-	-	1.77 ± 0.05	1.64 ± 0.06	1.93 ± 0.07**
Folate (μg/L)	9.24 ± 0.21	9.35 ± 0.23	8.46 ± 0.51	10.4 ± 0.34	10.4 ± 0.49	10.5 ± 0.46
Homocysteine (μmol/L)	9.98 ± 0.21	9.78 ± 0.21	11.53 ± 0.65**	4.74 ± 0.13	4.45 ± 0.17	5.11 ± 0.21*

*P* value compared to B_12_ (>190 ng/L) group; *P < 0.05; **P < 0.01. Continuous variables are mean ± SEM.

#### Saudi cohort

**Figure 1:**
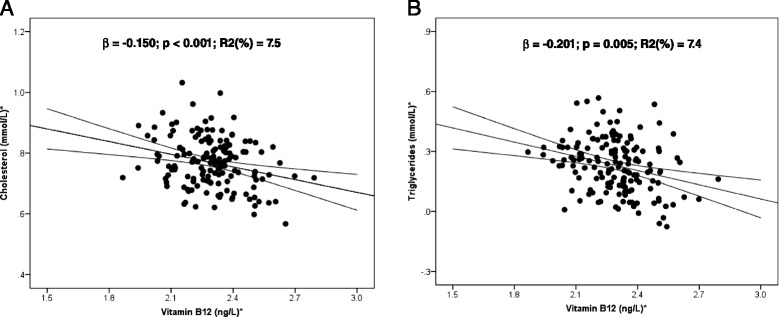
**Regression coefficient of vitamin B**_**12**_ **with (A) cholesterol and (B) triglycerides in pregnant women.** *Log-transformed for statistical comparisons. Model included B_12_, age, and BMI.

A cohort of 152 pregnant women living in Riyadh, KSA, participated in an observational study designed to assess the B_12_, folate, and homocysteine status during pregnancy. The data collection was carried out from pregnant women at the second trimester: between 16 to 28 weeks gestation. The prevalence of serum B_12_ insufficiency (<148 pmol/L) was 45%. Compared to normal B_12_ women, low B_12_ women had significantly higher BMI, cholesterol, triglycerides, and homocysteine than normal B_12_ women (Table 1). In addition, B_12_ independently predicted cholesterol (*β* = −0.150; *P* < 0.001) and triglycerides (*β* = −0.201; *P* = 0.005) after adjusting for age and BMI in multiple regression analysis. B_12_ explained 7.5% (*P* = 0.001) of the variation in cholesterol and 7.4% (*P* = 0.005) of the variation in triglycerides (Figure 1A and B).

### Low vitamin B_12_ induces cholesterol biosynthesis in human adipocytes

**Figure 2:**
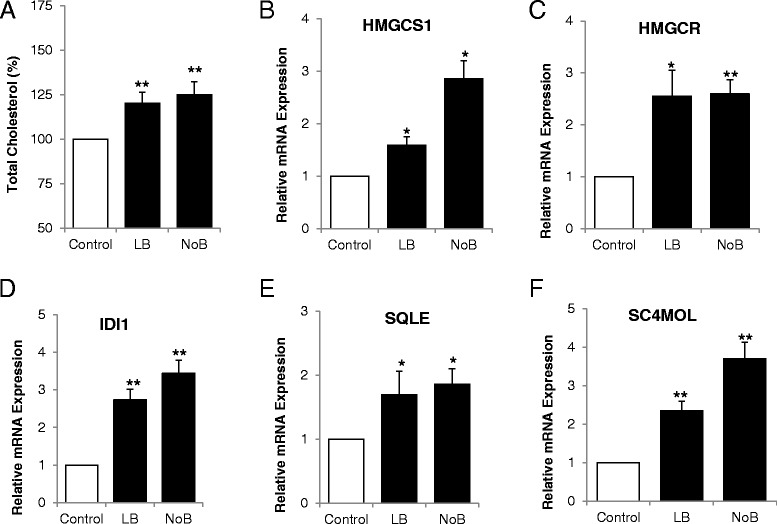
**Effect of vitamin B**_**12**_ **on cholesterol biosynthesis. (A)** Total cholesterol from adipocytes cultured in customised media: control (500 nM B_12_), LB (0.15 nM B_12_), and NoB (0 nM B_12_). Total cholesterol was normalized for protein content and expressed as percentage. mRNA expression of cholesterol biosynthesis genes in adipocytes. **(B)** HMGCS1, **(C)** HMGCR, **(D)** IDI1, **(E)** SQLE, and **(F)** SC4MOL. All experiments were performed as triplicates. Values are mean ± SEM. *P ≤ 0.05; **P ≤ 0.01, ***P ≤ 0.001, *P* value compared to control.

Human preadipocytes were cultured and fully differentiated in 12 different conditions representing varying concentrations of B_12_ and folic acid and intracellular cholesterol and extracellular homocysteine were measured. Adipocytes cultured in low (LB) or no B_12_ (NoB) media showed maximum and significant increase in total cholesterol and homocysteine levels compared with controls (Additional file 1: Figure S1 A and B). Folic acid alone had no significant effect on cholesterol and homocysteine levels. Therefore, we designed experiments to study only B_12_ deficiency while keeping the folic acid concentration constant to 6 μM for rest of the experiments and used three main conditions: (1) control (500 nM B_12_ represented adequate B_12_), (2) LB (0.15 nM B_12_ represented B_12_ deficiency) and (3) NoB (0 nM B_12_ represented extreme B_12_ deficiency). The total cholesterol was significantly increased in NoB and LB conditions compared to control (Figure 2A).

Expression of cholesterol biosynthesis genes was measured by qRT-PCR. In the first fundamental step, mevalonate formation from acetyl CoA, the expression of 3-hydroxy-3-methylglutaryl-CoA synthetase (HMGCS1) (Figure 2B; *P* < 0.001) and the rate limiting enzyme, 3-hydroxy-3-methylglutaryl-CoA reductase (HMGCR) (Figure 2C; *P* < 0.01) was significantly upregulated in NoB and LB condition compared to control. Similarly, the other downstream steps of this pathway showed the same pattern of significant gene expression changes. The other genes studied were isopentenyl diphosphate isomerase (IDI1), squalene epoxidase (SQLE) and methylsterol mono-oxygenase (SC4MOL) (Figure 2D,E,F; all *P* < 0.01).

### Low vitamin B_12_ induces expression of cholesterol synthesis and transport regulators

**Figure 3:**
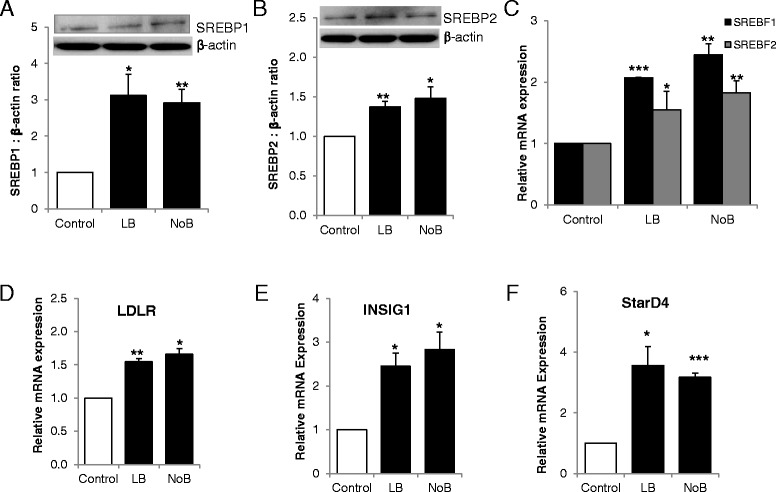
**Effect of vitamin B**_**12**_ **on regulation of cholesterol biosynthesis.** Protein and/or mRNA expression of genes regulating cholesterol biosynthesis in adipocytes. **(A)** SREBP-1, **(B)** SREBP-2, **(C)** SREBF1 and SREBF2, **(D)** LDLR, **(E)** INSIG1 and **(F)** StarD4. All experiments were performed as triplicates. Values are mean ± SEM. *P ≤ 0.05; **P ≤ 0.01, ***P ≤ 0.001, *P* value compared to control.

The sterol regulatory element-binding protein (SREBP-1 and 2) transcription factors and LDLR play a critical role in regulating cholesterol quantity. Protein as well as mRNA expressions of both SREBPs was significantly upregulated in NoB and LB conditions compared with control (Figure 3A,B,C; *P* < 0.05). The LDLR mRNA expression was also significantly higher in LB and NoB conditions compared with control (Figure 3D). The mRNA expression of other regulatory genes; insulin induced gene 1 (INSIG1) and StAR-related lipid transfer protein 4 (StarD4) was also significantly upregulated in both NoB and LB conditions compared to control (Figure 3E,F; both *P* < 0.01).

### SREBF1 and LDLR genes are hypomethylated in low vitamin B_12_ condition

Vitamin B_12_ plays an important role in regulating AdoMet levels which has been shown to regulate the SREBF1 expression [25]. AdoMet is the methyl donor and is responsible for methylation of DNA, protein, and lipids; we hypothesised that the upregulation of cholesterol synthesis in low B_12_ conditions could be due to altered methylation of genes regulating cholesterol biosynthesis. Therefore, we interrogated data from our genome-wide study to generate quantitative levels of DNA methylation at SREBF1, SREBF2, and LDLR.

**Figure 4:**
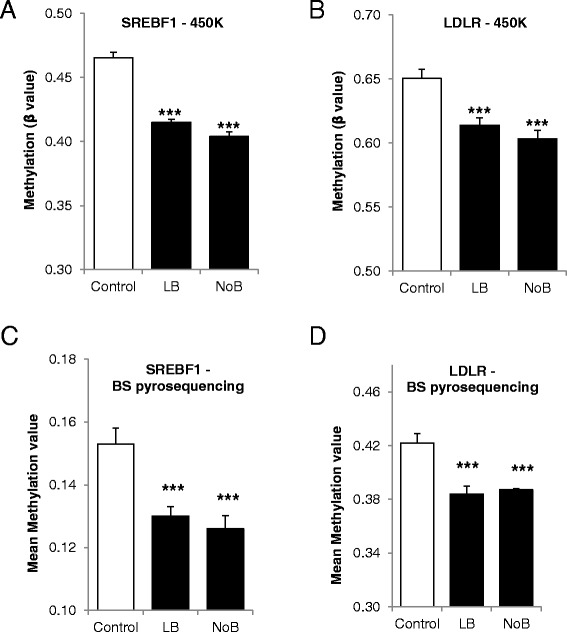
**Effect of vitamin B**_**12**_ **on methylation of cholesterol regulatory genes.** Genome-wide DNA methylation analysis of cholesterol regulatory genes was performed in adipocytes cultured in customised media, control, LB, and NoB. **(A)** SREBF-1 and **(B)** LDLR from Illumina. Validation by BS-pyrosequencing was also done in the same samples and methylation status confirmed for **(C)** SREBF-1 and **(D)** LDLR. All experiments were performed as triplicates. Values are mean ± SEM. *P ≤ 0.05; **P ≤ 0.01, ***P ≤ 0.001, *P* value compared to control.

Of the genes identified from the genome-wide dataset, probes at two targets; SREBF1 and LDLR showed differential methylation (reaching genome-wide significance) across experimental groups. The SREBF1 probe (cg27407935; chr17:17723235) showed a quantitative decrease in DNA methylation across from the control to LB to NoB, with mean *β* values for sample triplicates as follows: control, 0.47; LB, 0.41; NoB, 0.40 (*F* = 85.8, *P* value < 0.0001) (Figure 4A). This probe lies within the gene body of SREBF1 at a DNAse I hypersensitivity site and RNA Pol2 binding site (suggested by ENCODE data), suggesting a putative role in transcriptional regulation. (Additional file 1: Figure S4A). The LDLR probe (cg22971501; chr19:11199476) also showed relative hypomethylation across LBF and NoB with corresponding mean *β* values of 0.65, 0.61, and 0.60 (*F* = 14.41, *P* value < 0.0001) (Figure 4B). The LDLR probe lays 1,500 bp upstream of the 5′UTR at the gene promoter (Additional file 1: Figure S4B). For both SREBF1 and LDLR genes, the hypomethylation sites are close to the transcription start site (TSS) and are located near binding sites for PPARγ and C/EBPα (Additional file 1: Figure S4 A and B). These binding sites have been obtained from ChIP-seq data published elsewhere [28,29]. There was no significant difference in the mean beta values at SREBF2.

Validation experiments, using BS-pyrosequencing, confirmed the differential methylation at SREBF1 and LDLR between the experimental groups at the CpG sites defined by the methylation array probes. Mean methylation values for SREBF1 were as follows: control, 0.15; LB, 0.13; NoB, 0.13 (ANOVA, *P* value < 0.0001) (Figure 4C; Additional file 2: Table S3). Mean methylation values for LDLR were as follows: control, 0.42; LB, 0.38; NoB, 0.39 (ANOVA, *P* value < 0.0001) (Figure 4D; Additional file 2: Table S4). Between-group methylation differences validate those identified using the Illumina 450k array at the same CpG; the range of methylation values varies between BS-pyrosequencing and Illumina probe data due to the latter undergoing genome-wide normalisation and processing. The mRNA expression of both SREBF1 and LDLR was significantly increased in the LB and NoB conditions (Figure 3C,D; *P* < 0.01), same conditions in which they were hypomethylated.

### Vitamin B_12_ deficiency lowers AdoMet/AdoHcy

**Figure 5:**
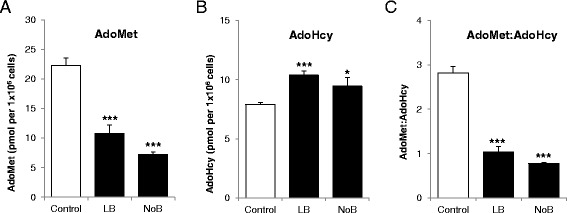
**Effect of vitamin B**_**12**_ **on methylation index.** AdoMet and AdoHcy was measured in cell pellets collected from the adipocytes cultured in customised media, control, LB, and NoB. **(A)** AdoMet, **(B)** AdoHcy, and **(C)** AdoMet-to-AdoHcy ratio. Normalized for cell number and expressed as pmol per 1 × 10^6^ cells. All experiments were performed as triplicates. Values are mean ± SEM. *P ≤ 0.05; **P ≤ 0.01, ***P ≤ 0.001, *P* value compared to control.

To test whether vitamin B_12_ deficiency impairs the cellular methylation potential (AdoMet/AdoHcy), intracellular AdoMet and AdoHcy were determined (Figure 5A,B). AdoMet and AdoHcy are the substrate and product of essential methyltransferase reactions. We found that adipocytes treated in B_12_ deficient conditions showed significantly decreased AdoMet, increased AdoHcy, and decreased AdoMet/AdoHcy (Figure 5C). A decrease in AdoMet/AdoHcy is predictive of reduced methylation potential (MP) and confirms that DNA methylation is likely to be affected in adipocytes cultured in deficient B_12_ conditions.

### Inhibition of methylation by 5-Aza-dC induces cholesterol biosynthesis but not homocysteine in high vitamin B_12_ condition

**Figure 6:**
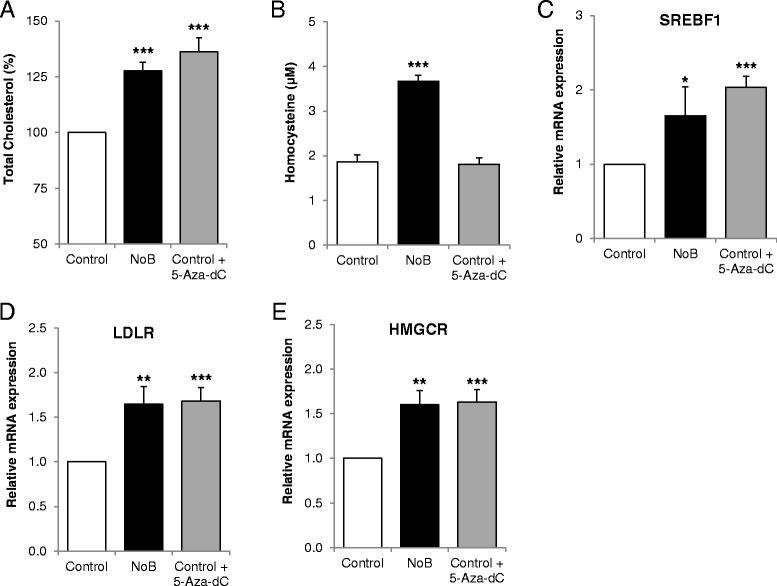
**Effect of vitamin B**_**12**_ **in the presence of methylation inhibitor. (A)** Total cholesterol in cell lysate. **(B)** Homocysteine in conditioned media. Relative mRNA expression of **(C)** SREBF1, **(D)** LDLR, and **(E)** HMGCR were measured in adipocytes maintained in customised media control, NoB, and control + 5-Aza-dC. 5-Aza-dC is methylation inhibitor. All experiments were performed as triplicates. Values are mean ± SEM. *P ≤ 0.05; **P ≤ 0.01, ***P ≤ 0.001, *P* value compared to control.

To further confirm that hypomethylation of SREBF1 and LDLR is the key mechanism by which cholesterol biosynthesis in human adipocytes is increased, we treated adipocytes with B_12_ in the presence of methylation inhibitor, 200 nM 5-Aza-dC (control + 5-Aza-dC). We observed that adipocytes treated with 5-Aza-dC showed significantly increased cholesterol accumulation compared to control (Figure 6A). There was no change in homocysteine levels under the same conditions (Figure 6B). This confirms that the increased cholesterol biosynthesis in B_12_-deficient conditions was due to reduced AdoMet/AdoHcy which resulted in hypomethylation of SREBF1 and LDLR. The expression of cholesterol biosynthetic gene HMGCR (Figure 6E) and cholesterol regulatory genes (SREBF1 and LDLR) (Figure 6C,D) was also significantly increased in NoB and control + 5-Aza-dC compared to control.

### B_12_ deficiency induces expression of cholesterol biosynthesis genes in human adipose tissue (UHCW cohort)

**Figure 7:**
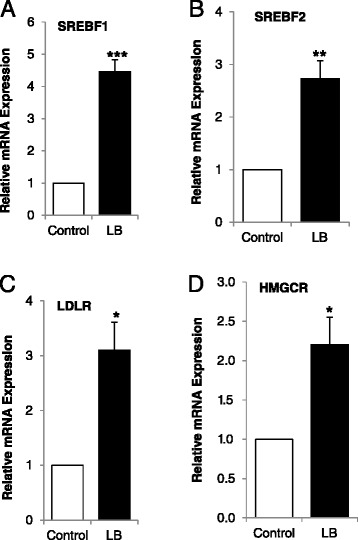
**Effect of vitamin B**_**12**_ **on human adipose tissue** ***ex vivo*** **.** mRNA expression of genes regulating cholesterol biosynthesis in adipocytes; **(A)** SREBF1, **(B)** SREBF2, **(C)** LDLR, and **(D)** HMGCR. Each group was a minimum of *n* = 5. Values are mean ± SEM. *P ≤ 0.05; **P ≤ 0.01, ***P ≤ 0.001, *P* value compared to control.

The prevalence of serum B_12_ deficiency in this cohort of pregnant women at the time of delivery was 39.6%. Low B_12_ mothers had significantly higher BMI, cholesterol, LDL cholesterol, triglycerides, and homocysteine than normal B_12_ mothers (Table 2). To validate the findings from our cultured primary human adipocytes, we further performed the key experiments in maternal subcutaneous adipose tissue (ScAT) chosen from normal B_12_ (*n* = 5) and low B_12_ (*n* = 5) mothers. We observed that the mRNA expressions of cholesterol regulatory genes; SREBF1, SREBF2 and LDLR (Figure 7A,B,C), and cholesterol biosynthetic gene; and HMGCR (Figure 7D) were upregulated in adipose tissue from low B_12_ mothers compared to normal B_12_ mothers (Figure 7).

**Table 2:** **Clinical characteristics of UHCW cohort**

**UHCW cohort**	**Total**	**Maternal B**_**12**_ **(ng/L)**	
	***n*** **= 91**	**>190 (** ***n*** **= 55)**	**≤190 (** ***n*** **= 36)**
Age (years)	32.7 ± 0.62	33.0 ± 0.84	32.3 ± 0.93
BMI (kg/m^2^)	29.4 ± 0.65	28.4 ± 0.82	30.8 ± 1.07*
Triglycerides (mmol/L)	2.69 (2.62, 3.06)	2.49 (2.37, 2.93)	3.04 (2.82, 3.48)*
Cholesterol (mmol/L)	6.48 (6.31, 6.89)	6.23 (5.99, 6.72)	6.86 (6.51, 7.43)*
LDL cholesterol (mmol/L)	3.53 (3.46, 3.95)	3.29 (3.16, 3.82)	3.91 (3.67, 4.38)*
HDL cholesterol (mmol/L)	1.56 (1.53, 1.72)	1.61 (1.54, 1.80)	1.49 (1.42, 1.70)
HOMA-IR	2.14 (2.47, 3.39)	1.87 (2.18, 3.51)	2.65 (2.45, 3.68)
Leptin (μg/L)	30.8 (32.3, 40.8)	29.3 (29.7, 40.6)	33.1 (31.3, 45.5)
Folate (μg/L)	10.5 (10.9, 13.2)	11.7 (11.5, 14.6)	9.0 (8.5, 12.4)*
Homocysteine (μmol/L)	6.23 (6.02, 7.54)	5.50 (5.26, 6.18)	7.53 (6.69, 10.11)**

Continuous variables are mean ± SEM or geometric mean (95% CI).

*P* value compared to maternal B_12_ (>190 ng/L) group; *P < 0.05; **P < 0.001.

### Effect of vitamin B_12_ in primary human adipocytes

In order to validate the findings from human adipocyte cell line ChubS-7, the key experiments were also performed in cultured primary human adipocytes under the same conditions. The total cholesterol as well as homocysteine was significantly increased in primary adipocytes in the LB and NoB conditions compared with control (Additional file 1: Figure S5 A and B). The mRNA expressions of genes regulating cholesterol biosynthesis, SREBF1 and SREBF2, and LDLR were also significantly increased (Additional file 1: Figure S5 C, D, and E).

## Discussion

The dietary supply of the methyl donors folic acid, vitamin B_12_, betaine, methionine, and choline is essential for normal growth, development, and physiological functions through the life course [19,30]. Both human [18,22,31,32] and animal [16] studies have shown that vitamin B_12_ deficiency is associated with altered lipid profile and metabolic disorder, and it has been hypothesized that this may be due DNA methylation changes. The results of the present study provide novel evidence that vitamin B_12_ plays a vital role in the biosynthesis of cholesterol through induction of SREBPs expression in human adipocytes and DNA hypomethylation due to limited availability of AdoMet. Specifically, we have demonstrated that low vitamin B_12_ (a) is associated and independently predicted higher total cholesterol, LDL cholesterol, and cholesterol-to-HDL ratio in serum; (b) induced cholesterol biosynthesis and homocysteine in adipocytes; (c) increased expression of SREBPs and genes responsible for cholesterol biosynthesis; (d) reduced AdoMet-to-AdoHcy levels and caused hypomethylation of SREBF1 and LDLR genes in their regulatory regions leading to increased mRNA expression; and (e) increased expression of SREBs, LDLR, and HMGCR in human adipose tissue.

Cholesterol is a vital component of all mammalian cells, and it is essential for normal functioning of the body; each cell synthesizes it from simpler molecules, a complex 37-step process that starts with the intracellular protein enzyme HMG-CoA reductase (HMGCR). However, high levels of cholesterol in the blood circulation, depending on how they are transported within lipoproteins, are strongly associated with increased risk of cardiovascular diseases (CVD) [33]. The causes of why some people are at higher risk of cholesterol dysregulation is not clear. Our study using three independent clinical cohorts: women at child bearing age, during early pregnancy, and at delivery shows that low vitamin B_12_ status was associated with higher total cholesterol, LDL cholesterol, and cholesterol-to-HDL ratio (Tables 1 and 2). A recent study in Europeans and Indians with type 2 diabetes found that vitamin B_12_ deficiency was associated with adverse lipid profile [34]. The first direct evidence that vitamin B_12_ deficiency could lead to adverse lipid profile has come from an *in vivo* study in Wistar rats [16] that showed offspring from vitamin B_12_-deficient mothers had higher adiposity and altered lipid metabolism. The authors of this study hypothesized that this was due to altered and dysfunctional adipocytes and showed that offspring from B_12_-deficient mothers had higher total cholesterol, triglycerides, IL-6, and TNF-α and had lower adiponectin and leptin compared with control offspring. To investigate the possible mechanisms underlying the findings of Kumar *et al*. [16], we chose human preadipocytes as model system as these cells undergo several population doublings before differentiation, mimicking the inter-generational effect in *in vitro* conditions. Upon investigation, we observed that the total intracellular cholesterol as well as extracellular homocysteine were significantly increased in low (LB) and no vitamin B_12_ (NoB) conditions, while folic acid levels had no significant effect either on homocysteine levels or on cholesterol (Additional file 1: Figure S1 A and B; Figure 1A). Though these pathways have previously been linked to hyperhomocysteinemia (HyHcy) [35], our data shows that low vitamin B_12_ independently contributes to both HyHcy and dysregulation of cholesterol synthesis. This provides the first mechanistic data in support of recent human cohort studies that identify an inverse relationship between vitamin B_12_ status and a phenotype of adiposity, gestational diabetes, T2D, and insulin resistance [17,18]. While the findings of these cohorts are only associations, a plausible causal biochemical mechanism is now proposed that can be explored further in longitudinal and intervention-based clinical studies.

The cholesterol biosynthesis pathway and regulation was investigated in detail and all the genes analyzed (Figure 2) were upregulated under vitamin B_12_-deficient conditions. The physiology of cholesterol and regulation has been reviewed elsewhere [7,36,37]. Most of the genes involved in cholesterol synthesis and transport are direct target of SREBPs. Activation of SREBPs is critical as they directly induce the expression of more than 30 genes dedicated to the synthesis, uptake, and control of the intracellular accumulation of cholesterol [6]. In this study, we observed that the protein and mRNA expressions of both SREBPs were significantly increased in vitamin B_12_-deficient conditions. Regulation of cholesterol biosynthesis is complex, and we observed increased expression of other regulatory genes under the same conditions, LDLR, INSIG1, and StarD4. LDLR along with SREBP’s plays a critical role in regulating the amount of cholesterol (Figure 3). The INSIG1 encodes an ER membrane protein that plays a critical role in regulating cholesterol concentrations in cells and directly interacts with SREBP2 [38,39]. StarD4 has specifically been linked to the movement of cholesterol to the ER [40]. Increases in levels of either SREBP-1 or SREBP2 increases StarD4 levels in mouse liver, and increased StarD4 increases active SREBP2 levels [41].

The close relationship of B_12_, folic acid, and homocysteine in the one-carbon cycle plays an important role in regulating the levels of AdoMet and AdoHcy [26,27]. AdoMet is used in the methylation of DNA, RNA, proteins, phospholipids, and a number of other small molecules and the ratio between AdoMet and AdoHcy may be crucial in this process (reviewed [26]). Using TCblR/CD320 knockout mice, Fernandez-Roig *et al*. [23] have shown that vitamin B_12_ deficiency led to DNA hypomethylation in the brain. We observed that the two cholesterol regulating genes, SREBF1 and LDLR, were significantly hypomethylated in their regulatory regions in the cells cultured in low or no B_12_ media compared with the cells cultured control media which has high B_12_ (Figure 4). Walker *et al*. [25] have noted that restricting AdoMet caused increased SREBP1-dependent transcription and lipid accumulation. We measured the AdoMet-to-AdoHcy ratio and found it to be significantly lower in LB and NoB condition compared with controls confirming the restricted availability of AdoMet in a B_12_-deficient environment (Figure 5). The AdoMet-to-AdoHcy ratio is known as a marker of DNA MP due its direct relationship to the availability of methyl groups capable of methylation. We confirmed DNA hypomethylation in these conditions in both SREBF1 and LDLR, and that the sites of hypomethylation were close to the TSS and were located near binding sites for PPARγ and C/EBPα, master regulators of lipogenesis and adipocyte differentiation (Additional file 1: Figure S4 A and B). These binding sites in human adipocytes were obtained from ChIP-seq study published by Mikkelsen *et al*. [28]. The significance of this observation was further enhanced as the mRNA expression, of these two genes, were also increased under the same conditions. This is one of the first observations where gene specific altered methylation has been observed due to specific nutrient deficiency in a human cell type.

To confirm that cholesterol biosynthesis was induced due to change in methylation status of SREBF1 and LDLR in B_12_ restricted conditions, we cultured adipocytes in control media with methylation inhibitor 5-Aza-dC and measured total cholesterol and homocysteine. Interestingly, the total cholesterol was higher in control + 5-Aza-dC cells compared to control (Figure 6A), similar to cells cultured in NoB condition but found that methylation inhibition had no effect on homocysteine levels (Figure 6B). The mRNA expression of SREBF1, LDLR, and HMGCR was also significantly increased in the control + 5-Aza-dC condition compared with control (Figure 6C,D,E). This further confirmed that the increased cholesterol biosynthesis in vitamin B_12_-deficient conditions was due to reduced AdoMet/AdoHcy levels, not high homocysteine, resulting in hypomethylation of SREBF1 and LDLR. To further confirm the effect of vitamin B_12_ in cholesterol biosynthesis *ex vivo*, we used human adipose tissue from vitamin B_12_ deficient and sufficient mothers undergoing caesarean section. Intriguingly, we found that the gene expressions of cholesterol regulatory genes (SREBF1, SREBF2, LDLR) and cholesterol biosynthetic gene HMGCR were significantly upregulated in low vitamin B_12_ group compared to control (Figure 7). In the same cohort, low vitamin B_12_ women had significantly higher BMI, cholesterol, LDL cholesterol, triglycerides, and homocysteine than normal vitamin B_12_ women (Table 2), supporting the findings of Kumar *et al*. [16] in their animal model. Our human data suggest that vitamin B_12_ deficiency could alter certain pathways and may play an important role in predicting future metabolic risk.

Adipose tissue is the largest pool of free cholesterol in the body [42,43]. In rats, a decreased intracellular cholesterol content and simultaneous increase in LDL-C levels have been observed under fasting conditions [4,42,44,45]. In contrast, an elevation of the adipocyte cholesterol amount has also been reported in obese rodents and humans [43,46] and studies using ob/ob mice show increased HMGCR activity in adipose (but not liver) and higher plasma cholesterol in young obese mice compared to control littermates, suggesting an adipocyte-specific regulation of cholesterol metabolism in obesity [8,47]. Our clinical observations show strong associations of vitamin B_12_ deficiency with BMI, triglycerides, and total cholesterol, and our *in vitro* studies in adipocytes show that vitamin B_12_-deficient conditions induce *de novo* cholesterol biosynthesis. Studies have also highlighted a strong association between adipocyte cholesterol content and fat cell size and triglyceride content and bigger the fat cell, the more cholesterol it has been shown to contain [8,13,47]. The rate of *de novo* cholesterol synthesis in adipose tissue is only 4% compared to that of liver [44] and therefore, may not contribute significantly to the circulatory cholesterol but current literature suggests an important role for adipose tissue in regulation of circulatory HDL-C by mediating HDL lipidation [2,48]. Chung *et al*. [2] have shown that specific deletion of cholesterol transporter ABCA1 in adipose tissue significantly reduces HDL-C levels. The increased biosynthesis in adipose tissue therefore, may interfere with the lipidation process in vitamin B_12_-restricted conditions. The aim of this manuscript is to show how specific pathways could be regulated by vitamin B_12_ and provide a direct mechanism through its role in regulating AdoMet levels. Further, *in vivo* studies are required to measure contribution of adipose tissue in circulatory cholesterol, particularly under vitamin B_12_-deficient conditions.

## Conclusions

In conclusion, the vitamin B_12_ plays an important role in the adipocyte metabolism and its deficiency led to increased homocysteine and total cholesterol. This is one of the first studies providing a direct mechanism by which vitamin B_12_ may be regulating wider metabolic changes, particularly in states of deficiency. Our findings highlight the importance of adequate levels of vitamin B_12_ during the periconceptional period and the relevance to groups prone to deficiency such as vegetarians and vegans. There may also be a case for how we diagnose vitamin B_12_ deficiency which can be improved by measurement of the active plasma fraction of transcobalamin (holo-TC) [49], based on our *in vitro* observations where the effect of vitamin B_12_ deficiency was much more severe compared to clinical observations. Understanding the complete mechanisms of the role of vitamin B_12_ on other tissue systems will likely provide further insights on metabolic programming.

## Methods

### Clinical cohorts

Data from two independent clinical cohorts was used.

#### NDNS cohort

NDNS survey was conducted between July 2000 and June 2001, in a nationwide representative sample of 3,704 patients across the country. Of these, 1,256 subjects had full biochemical analysis which included lipid profiles, B_12_, folic acid, and homocysteine and were used for the analysis

#### Saudi pregnant women cohort

This cohort consisted of 152 pregnant women living in Riyadh, KSA, who participated in an observational study designed to assess the vitamin B_12_, folate, and homocysteine status during pregnancy. Pregnant women attending the antenatal clinics, primary health clinics, and the Prince Salman Bin Abdulaziz Hospital in the western health sector in Riyadh were recruited. Participating subjects were asked to fast before their visit to be enrolled in the study. Women aged 18 to 40 years, non-smokers, with pre-pregnancy BMI > 17 and <37 kg/m^2^, without any significant medical problems, and not under any medication were eligible for the study. Women were excluded if they were anemic, on multivitamins, or had any nutrition-related pre-existing medical condition such as coeliac disease, known diabetes or any liver, cardiac, or kidney diseases. The data collection was carried out from May 2009 to December 2010 from pregnant women at the second trimester; between 16 and 28 weeks gestation.

#### University Hospital Coventry and Warwickshire (UHCW) cohort and adipose tissue collection

Women with singleton pregnancy undergoing elective caesarean section at term (37 to 42 weeks of gestation) were recruited after written informed consent (UHCW cohort). Women with known chronic diseases were excluded. Maternal anthropologic measures, data including parity, smoking, diet, and vitamin use were collected. Paired maternal venous blood samples (*n* = 91) and adipose tissue (subcutaneous) were collected at the time of caesarean section. Fasting maternal blood and cord blood samples collected in tubes without anticoagulant were centrifuged at 2,000 rpm/10 min. Serum was separated, aliquoted, and stored at −80°C until analysis. All tissue samples were flash frozen and/or utilized for characterizing gene expression and protein expression by RNA isolation and protein extraction, respectively. The study was approved by the NHS Research Ethics Committee (07/H1210/141).

### Culture and differentiation of human pre-adipocytes

Human preadipocyte cell line Chub-S7 [50] and human primary preadipocytes were cultured and differentiated as described elsewhere [51]. Briefly, the preadipocytes were grown to confluence in customised growth media (GM). At confluence (day 0), preadipocytes were differentiated in differentiation media (DM) for 72 h (day 03) and were maintained in nutrition media (NM) thereafter for 11 days (day 14). The day differentiation was initiated (at confluence) was considered as day 0. On day 14, conditioned media were collected and cells were harvested for protein and RNA analysis and stored at −80°C until use. Primary human preadipocytes were isolated from human abdominal subcutaneous (AbSc) AT, collected from patients (age: 40.8 ± 5 years; lean BMI: 22.04 ± 2.6 kg/m^2^) undergoing surgery with informed consent obtained in accordance with LREC guidelines and with ethics committee approval.

The DMEM/F-12 medium used above was customised initially for 12 different B_12_ and folic acid concentrations (Additional file 1: Figure S1) but we will present three main conditions as follows: (1) control: normal B_12_ (B_12_, 500 nM; folic acid, 6 μM); (2) LB: low B_12_ (B_12_, 0.15 nM; folic acid, 6 μM); and (3) NoB: no B_12_ and 6 μM folic acid. For methylation inhibition, preadipocytes were incubated with 200 nM of 5-aza-2′-deoxycytidine (5-Aza-dC) at 50% confluency for 72 h and were differentiated at confluence.

### Isolation of RNA and quantitative real time-PCR (qRT-PCR) analysis

The total RNA from the adipocytes was isolated using RNeasy Lipid Tissue Kit (Qiagen, Venlo, Limburg) according to manufacturer’s protocol. cDNA synthesis, quantitative PCR and analysis were conducted as described elsewhere [51]. RNA expressions were normalized to the housekeeping gene 18s rRNA (Applied Biosystems, Paisley, UK, 4319413E). The custom taqman gene expression assays from Applied Biosystems were used for all the genes; their catalogue numbers are provided in brackets. The details of the gene assays are: SREBF1 (Hs01088691_m1); SREBF2 (Hs01081784_m1); LDLR (Hs00181192_m1); HMGCR (Hs00168352_m1); HMGCS1 (Hs00940429_m1); IDI1 (Hs01057440_m1); SQLE (Hs01123768_m1); SC4MOL (Hs00932159_m1); Insig1 (Hs01650979_m1); and StarD4 (Hs00287823_m1).

### Western blot analysis

Cells were washed with ice cold phosphate-buffered saline (PBS) and harvested in lysis buffer (radioimmunoprecipitation assay (RIPA) buffer with protease and phosphatase inhibitors) and stored at −80°C until required [51]. Extracts were quantitated by the Bradford protein assay. Western blot analysis was performed as described elsewhere [52].

### Measurement of cholesterol and homocysteine

Total cholesterol was determined fluorimetrically in cell lysates using Amplex Red cholesterol assay kit according to manufacturer’s protocol (Invitrogen, Carlsbad, CA, USA). Reduced homocysteine was chromatographically separated using ion-paired reversed-phase HPLC with oxidative mode electrochemical detection (ECD). Conditioned media or serum was reduced with 50 mM DTT at 37°C for 15 min, precipitated by 150 g/L sulphosalicylic acid and centrifuged and supernatant used for detection and analysis.

### DNA methylation

Genome-wide DNA methylation was performed using Illumina HumanMethylation 450Beadchip (Illumina, San Diego, CA, USA) on sample triplicates from the control, LB, and no B groups. High-quality genomic DNA was bisulphite converted using EZ-96 DNA Methylation Kit (Zymo Research, Irvine, CA, USA) according to manufacturer’s protocols. Efficiency of the C-to-T bisulphite conversion was tested using qPCR using primers to regions of the MLH and GAPDH genes (see Additional file 2: Table S1). Samples showing high (>98%) conversion efficiency were hybridised to the array. Arrays were scanned using two-color laser scanner. Data was quantile-normalized (per probe and per color channel). An R-based Limma package available via Bioconductor 2.11 was used to generate quantitative methylation values (*β*-values) to identify regions of differential methylation.

Validation of the relevant regions of differential methylation was performed using bisulphite-pyrosequencing, a quantitative assay of DNA methylation with single base pair resolution. Bisulphite converted DNA was amplified using primers designed to the relevant target regions (see Additional file 2: Methods) and pyrosequencing performed using Qiagen PyroMark software and system.

### AdoMet/s-adenosylhomocysteine (AdoHcy)

Measurement of intracellular AdoMet and AdoHcy were determined by stable isotopic dilution analysis liquid chromatography using a Waters Acquity UPLC system with a Quattro Premier XE tandem mass spectrometric detector (Waters Corporation, Milford, MA, USA). Briefly, isotopic standard (AdoMet-d3/AdoHcy-d4; 2 pmol/μl) was added to the cell pellets, deproteinized by 5% TCA and centrifuged at 6,000 *g* for 5 min at 4°C. The supernatant was then filtered and the ultrafiltrate was analyzed by LC-MS/MS system [53].

### Measurement of 2-deoxy-D-[1-3H] glucose uptake

Cells were washed with PBS and incubated at 37°C for 3 h in KRH buffer plus 0.01% BSA and 5 mmol/L glucose for insulin and serum starvation. Then, adipocytes were pre-incubated for 30 min at 37°C with KRH buffer without glucose with insulin, 1 μCi of 2-deoxy-D-[1-3H]glucose/ml was added and incubated for further 10 min. Cells were washed with ice-cold PBS and harvested in RIPA. Radioactivity was counted after adding scintillation fluid.

### Analytical determinations for clinical study

Serum, HDL and LDL cholesterol, and triglycerides were determined using an auto analyzer Synchron CX7 (Beckman Coulter, Brea, CA, USA) based on enzymatic colorimetric assays. B_12_ and folate were determined by electrochemiluminescent immunoassay using a Roche Cobas immunoassay analyzer. The reference values in our laboratory for non-pregnant women are as follows: 191 to 663 ng/L for vitamin B_12_ and 4.6 to 18.7 μg/L for folate.

### Statistical analysis

Laboratory data is presented as mean ± standard error of the mean (SEM) for at least three independent experiments to ensure reproducibility. Student’s *t*-test was used to compare values between two groups unless stated otherwise. *P* values < 0.05 were considered statistically significant. For clinical data, the biochemical parameters were log-transformed before analysis, as they were not normally distributed. All data were presented as mean ± standard error. No imputation was performed for the missing data. Means of continuous variables were compared using independent *t*-tests. Bivariate correlations between different variables were done using Spearman’s test. To assess the independent role of B_12_ on outcome variables, multivariate linear regression was carried out, adjusting for all possible confounders including age and BMI as independent variables. Care was taken to avoid inter-correlated variables in the regression analyses. For all the tests, *P* values < 0.05 were considered to be statistically significant. All analyses were performed using IBM SPSS Statistics version 19.

## Additional files

Additional file 1: Figure S1. Effect of B_12_/folic acid on total cholesterol and homocysteine (Hcy) levels. (A) Total intracellular cholesterol from cell lysates and (B) extracellular levels of Hcy from conditioned media measured from the adipocytes cultured in customised media supplemented with B_12_ (0 to 500 nM) and folic acid (0 to 60 μM). All experiments were performed as triplicates. Values are mean ± SEM. **P* ≤ 0.05; ***P* ≤ 0.01, ****P* ≤ 0.001, *P* value compared to control. **Figure S2.** Bisulphite-pyrosequencing amplicon; (A) SREBF1 - located on chromosome 17,723,166-17,723,321. (B) LDLR - located on chromosome 19; 11,199,399-11,199,590. Unconverted sequence is shown. Flanking primer sequences for the BS-pyrosequencing amplicon are marked in green and the sequencing primer is in yellow. This region contains CpG sites (in red and numbered in sequencing order). **Figure S3.** Location of Illumina 450k probe and bisulphite pyrosequencing assay: (A) SREBF1 - cg27407935 and (B) LDLR-cg22971501. **Figure S4.** Transcript variants of (A) SREBF1 and (B) LDLR, plotted using UCSC ENCODE browser, showing location markers for H3K27Ac, DNaseI hypersensitivity, transcription factor binding density across ChIP-Seq experiments and CpG islands. Transcription start sites have been added, indicated in red arrows above, and PPARγ gamma and C/EBPα binding sites, assayed by ChIP-Seq. in human adipocytes [32,33] indicated in red rectangles below. The sites we found to be significantly differentially methylated are indicated by yellow stars. **Figure S5.** Effect of B_12_ in primary adipocytes: (A) Total cholesterol was measured fluorimetrically in cell lysates collected from the differentiated Chub-S7 adipocytes maintained in customised media supplemented with B_12_ (0, 0.15, 500 nM) and folate (6 μM) concentrations. Total cholesterol was normalized for protein content and expressed as percentage. (B) Homocysteine in conditioned media. Relative mRNA expression of (C) SREBF1, (D) SREBF2, and (E) LDLR were measured from the differentiated Chub-S7 adipocytes maintained in customised media supplemented with B_12_ (0, 0.15, 500 nM) and folate (6 μM) concentrations. All experiments were performed as triplicates. Values are mean ± SEM. **P* ≤ 0.05; ***P* ≤ 0.01, ****P* ≤ 0.001, *P* value compared to control. Additional file 2: Table S1. Primer Sequences for bisulphite conversion efficiency experiments. **Table S2.** Bisulphite-pyrosequencing primer design for LDLR and SREBF1. **Table S3.** Methylation values per CpG within the SREBF1 amplicon measured by bisulphite-pyrosequencing. CpG 1 (in bold) denotes the cg2747935 Illumina 450k probe showing genome-wide significance between experimental groups. **Table S4.** Methylation values per CpG within the LDLR amplicon measured by bisulphite-pyrosequencing. CpG 2 (in bold) denotes the cg22971501 Illumina 450k probe showing genome-wide significance between experimental groups. No data was generated from CpG 4 due to poor sequencing efficiency. **Table S5.** Beta (methylation)-values at all Illumina 450k probes per experimental group located within the LDLR, SREBF1 and SREBF2 genes. The two probes where between-group methylation difference reaches genome-wide significance (FDR-adjusted *P* value < 0.05) are highlighted in yellow.
