# Supplementary material for: Vitamin B12 insufficiency induces cholesterol biosynthesis by limiting s-adenosylmethionine and modulating the methylation of SREBF1 and LDLR genes
Source: Clin Epigenetics. 2015 Feb 27;7(1):14. doi: 10.1186/s13148-015-0046-8 (PMC4356060; doi:10.1186/s13148-015-0046-8)
Supplement: Additional file 1: Figure S1. — Effect of B12/folic acid on total cholesterol and homocysteine (Hcy) levels. (A) Total intracellular cholesterol from cell lysates and (B) extracellular levels of Hcy from conditioned media measured from the adipocytes cultured in customised media supplemented with B12 (0 to 500 nM) and folic acid (0 to 60 μM). All experiments were performed as triplicates. Values are mean ± SEM. *P ≤ 0.05; **P ≤ 0.01, ***P ≤ 0.001, P value compared to control. Figure S2. Bisulphite-pyrosequencing amplicon; (A) SREBF1 - located on chromosome 17,723,166-17,723,321. (B) LDLR - located on chromosome 19; 11,199,399-11,199,590. Unconverted sequence is shown. Flanking primer sequences for the BS-pyrosequencing amplicon are marked in green and the sequencing primer is in yellow. This region contains CpG sites (in red and numbered in sequencing order). Figure S3. Location of Illumina 450k probe and bisulphite pyrosequencing assay: (A) SREBF1 - cg27407935 and (B) LDLR-cg22971501. Figure S4. Transcript variants of (A) SREBF1 and (B) LDLR, plotted using UCSC ENCODE browser, showing location markers for H3K27Ac, DNaseI hypersensitivity, transcription factor binding density across ChIP-Seq experiments and CpG islands. Transcription start sites have been added, indicated in red arrows above, and PPARγ gamma and C/EBPα binding sites, assayed by ChIP-Seq. in human adipocytes [32,33] indicated in red rectangles below. The sites we found to be significantly differentially methylated are indicated by yellow stars. Figure S5. Effect of B12 in primary adipocytes: (A) Total cholesterol was measured fluorimetrically in cell lysates collected from the differentiated Chub-S7 adipocytes maintained in customised media supplemented with B12 (0, 0.15, 500 nM) and folate (6 μM) concentrations. Total cholesterol was normalized for protein content and expressed as percentage. (B) Homocysteine in conditioned media. Relative mRNA expression of (C) SREBF1, (D) SREBF2, and (E) LDLR were measured from the differentiate [file 13148_2015_46_MOESM1_ESM.pptx]

## Slide 1
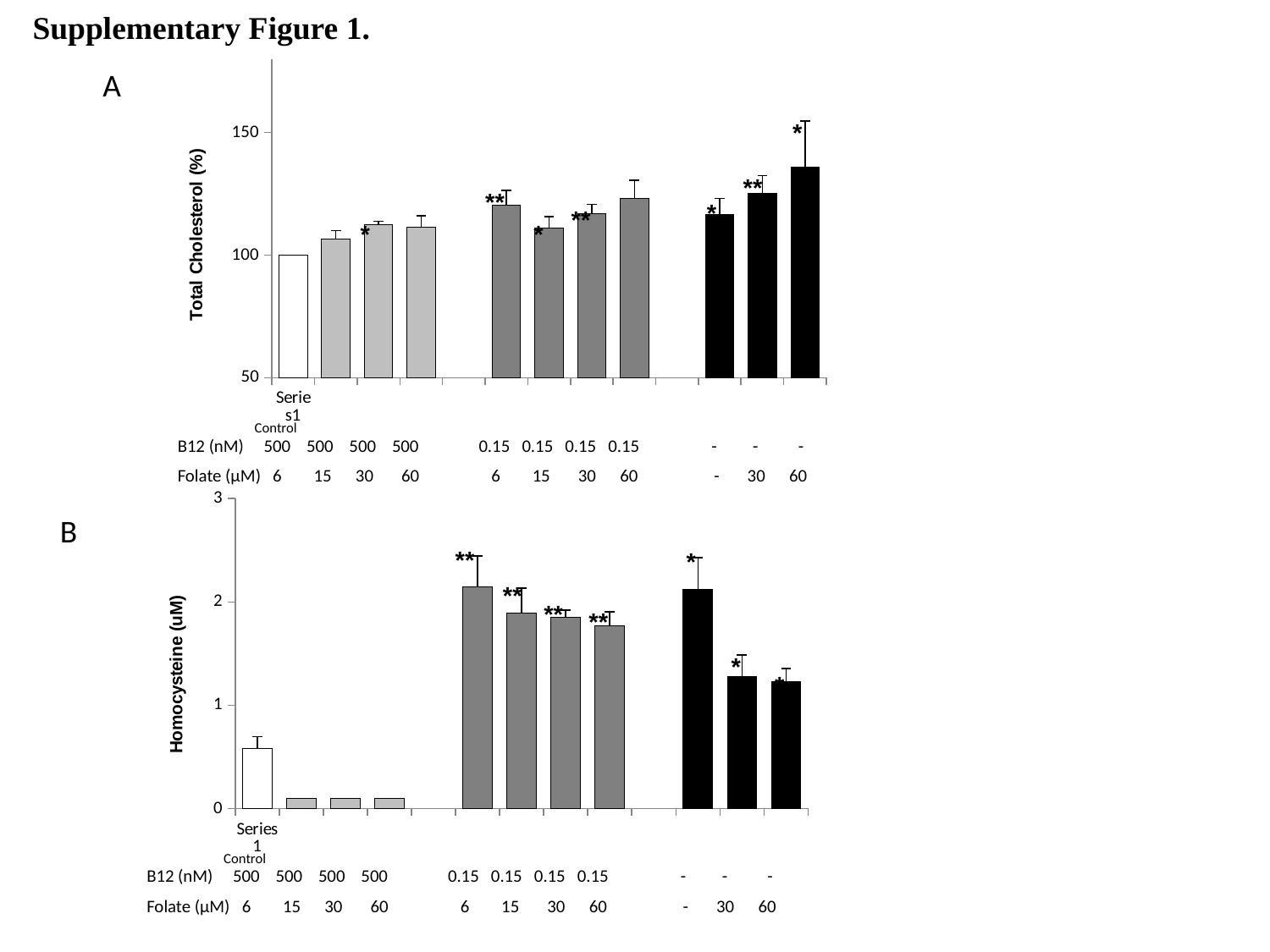

Supplementary Figure 1.
### Chart
| Category | |
|---|---|
| | 100.0 |
| | 106.6473728820329 |
| | 112.3333226875807 |
| | 111.4838591961181 |
| | None |
| | 120.2551175502319 |
| | 111.1534754783535 |
| | 116.9719246047082 |
| | 123.0097189730295 |
| | None |
| | 116.5945476944001 |
| | 125.1208061740556 |
| | 136.1011432658997 |*
*
**
**
*
*
**
Control
B12 (nM) 500 500 500 500 0.15 0.15 0.15 0.15 - - -
Folate (µM) 6 15 30 60 6 15 30 60 - 30 60
A
### Chart
| Category | |
|---|---|
| | 0.578888888888889 |
| | 0.1 |
| | 0.1 |
| | 0.1 |
| | None |
| | 2.143333333333334 |
| | 1.89 |
| | 1.853333333333333 |
| | 1.773333333333333 |
| | None |
| | 2.118888888888887 |
| | 1.276666666666667 |
| | 1.232222222222222 |*
**
**
**
*
*
**
Control
B12 (nM) 500 500 500 500 0.15 0.15 0.15 0.15 - - -
Folate (µM) 6 15 30 60 6 15 30 60 - 30 60
B
*

## Slide 2
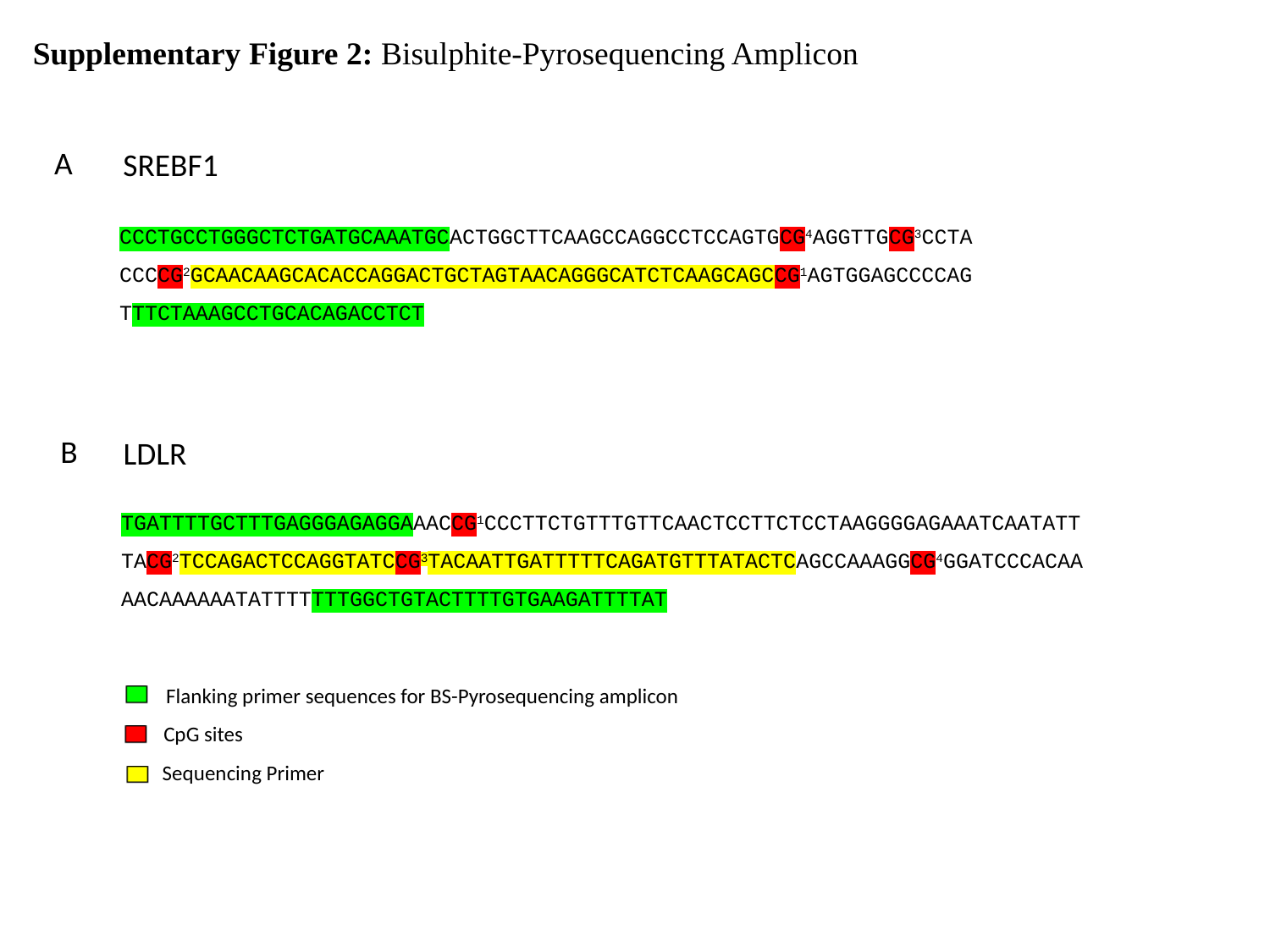

Supplementary Figure 2: Bisulphite-Pyrosequencing Amplicon
A
SREBF1
CCCTGCCTGGGCTCTGATGCAAATGCACTGGCTTCAAGCCAGGCCTCCAGTGCG4AGGTTGCG3CCTACCCCG2GCAACAAGCACACCAGGACTGCTAGTAACAGGGCATCTCAAGCAGCCG1AGTGGAGCCCCAGTTTCTAAAGCCTGCACAGACCTCT
B
LDLR
TGATTTTGCTTTGAGGGAGAGGAAACCG1CCCTTCTGTTTGTTCAACTCCTTCTCCTAAGGGGAGAAATCAATATTTACG2TCCAGACTCCAGGTATCCG3TACAATTGATTTTTCAGATGTTTATACTCAGCCAAAGGCG4GGATCCCACAAAACAAAAAATATTTTTTTGGCTGTACTTTTGTGAAGATTTTAT
Flanking primer sequences for BS-Pyrosequencing amplicon
CpG sites
Sequencing Primer

## Slide 3
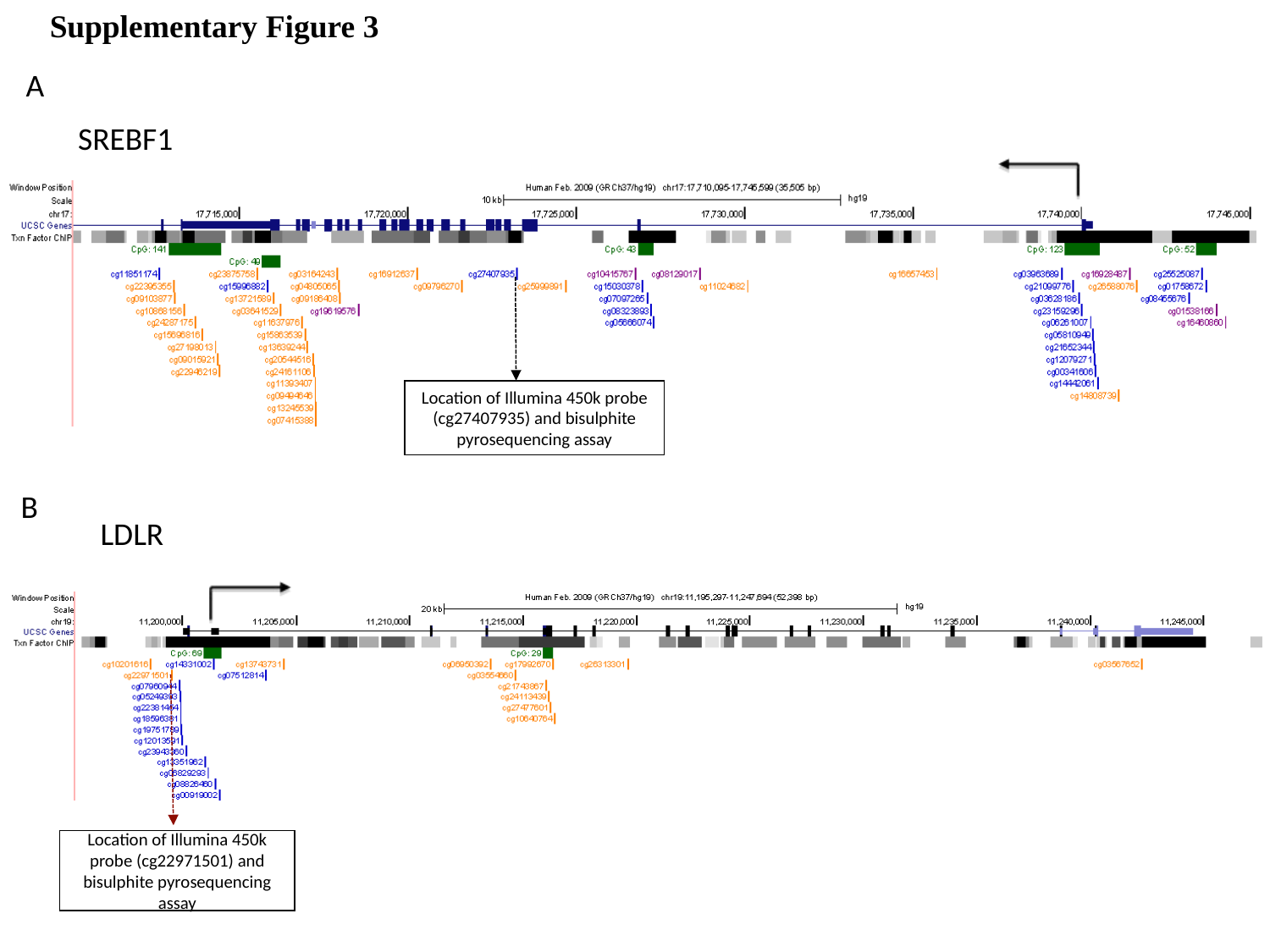

Supplementary Figure 3
A
SREBF1
Location of Illumina 450k probe (cg27407935) and bisulphite pyrosequencing assay
B
LDLR
Location of Illumina 450k probe (cg22971501) and bisulphite pyrosequencing assay

## Slide 4
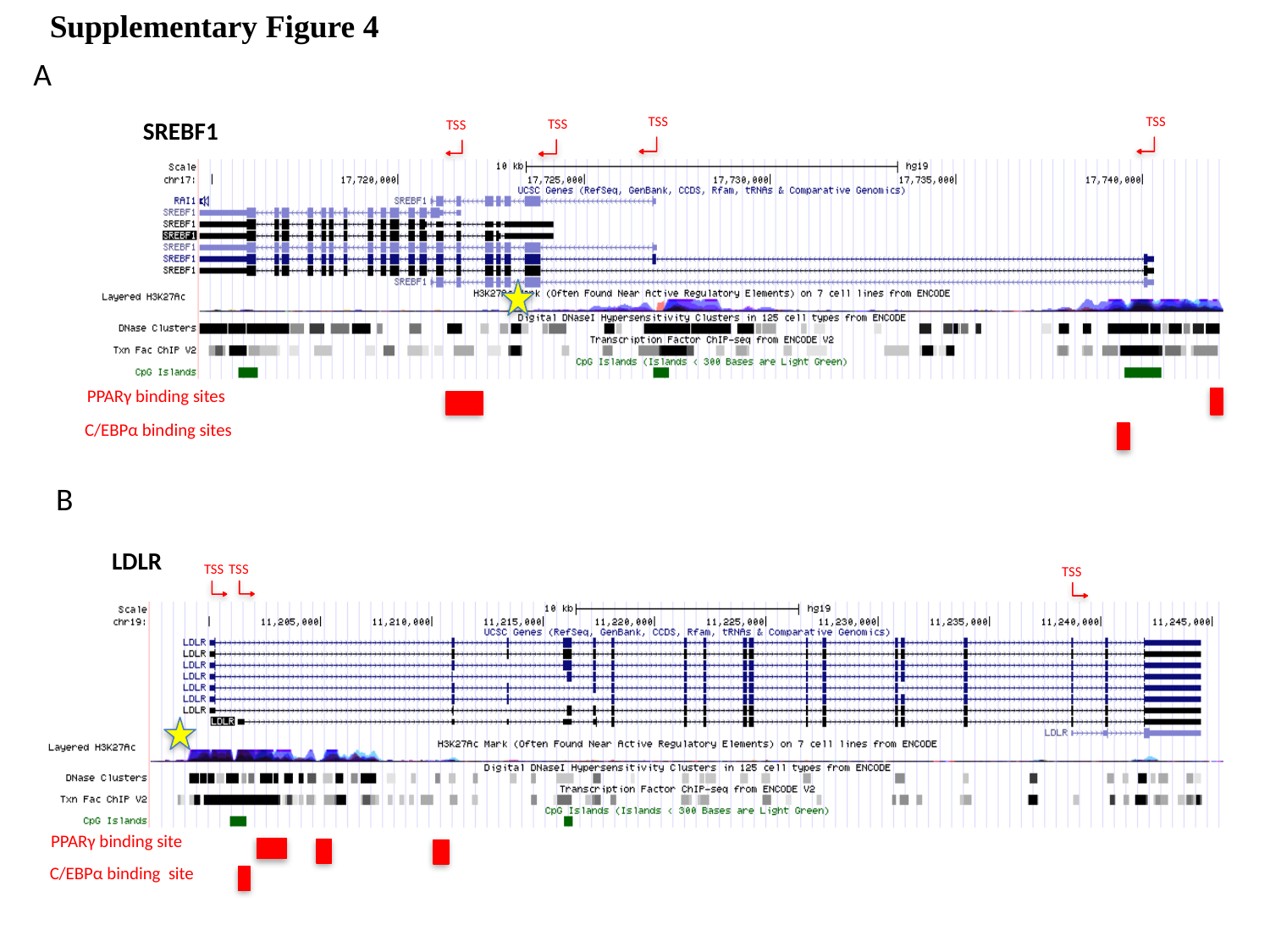

Supplementary Figure 4
A
TSS
TSS
SREBF1
PPARγ binding sites
C/EBPα binding sites
TSS
TSS
B
LDLR
TSS
PPARγ binding site
C/EBPα binding site
TSS
TSS

## Slide 5
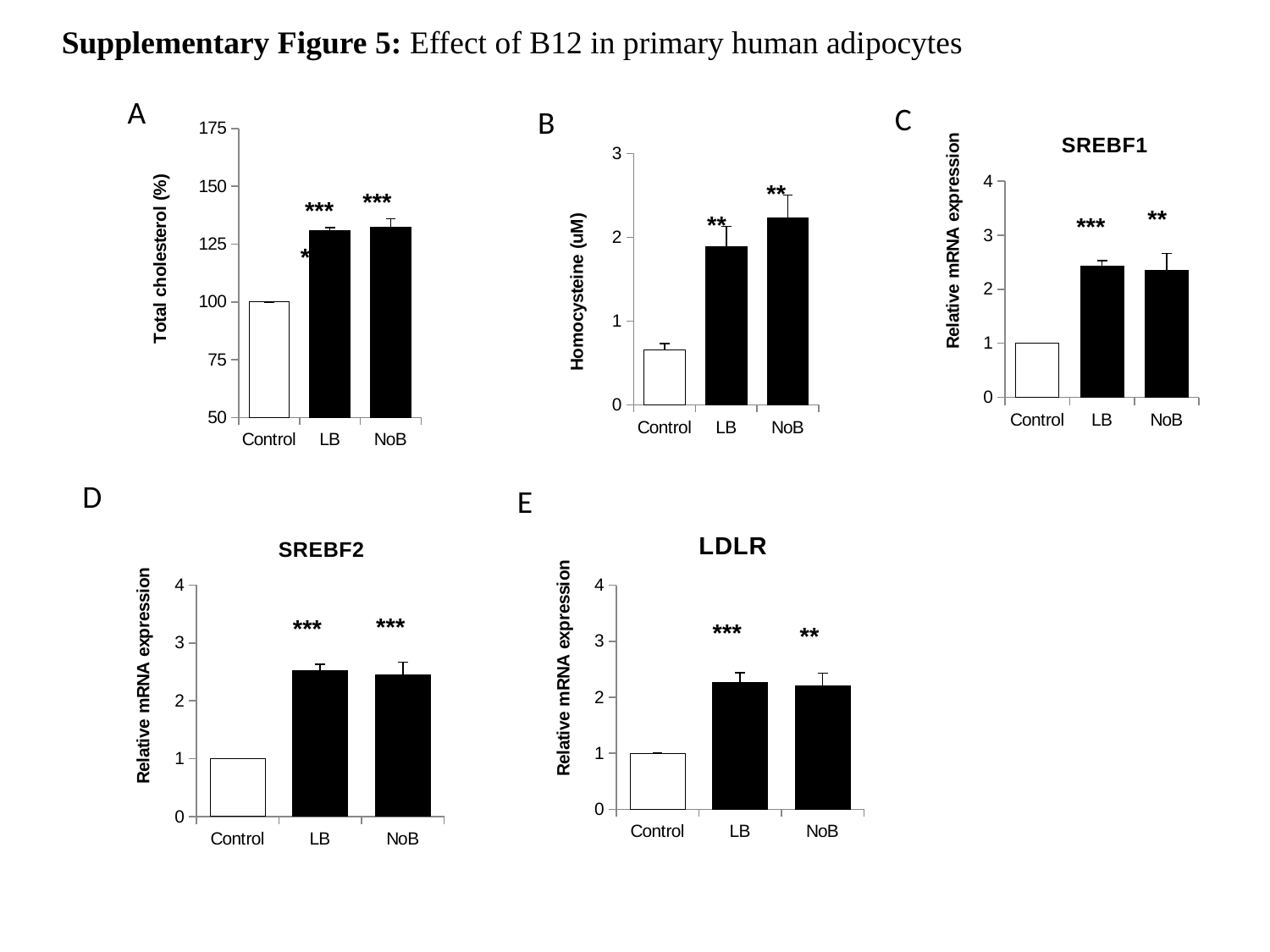

Supplementary Figure 5: Effect of B12 in primary human adipocytes
A
C
B
### Chart
| Category | |
|---|---|
| Control | 100.0 |
| LB | 130.7293651409418 |
| NoB | 132.3130330965379 |***
**
***
### Chart: SREBF1
| Category | |
|---|---|
| Control | 1.0 |
| LB | 2.433572823652188 |
| NoB | 2.352138013151027 |**
***
### Chart
| Category | |
|---|---|
| Control | 0.652 |
| LB | 1.89 |
| NoB | 2.23 |**
**
D
E
### Chart: LDLR
| Category | |
|---|---|
| Control | 1.0 |
| LB | 2.26962034498895 |
| NoB | 2.198043066520725 |***
**
### Chart: SREBF2
| Category | |
|---|---|
| Control | 1.0 |
| LB | 2.52072881323339 |
| NoB | 2.442659337985637 |***
***
