# Supplementary material for: Vitamin B12 insufficiency induces cholesterol biosynthesis by limiting s-adenosylmethionine and modulating the methylation of SREBF1 and LDLR genes
Source: Clin Epigenetics. 2015 Feb 27;7(1):14. doi: 10.1186/s13148-015-0046-8 (PMC4356060; doi:10.1186/s13148-015-0046-8)
Supplement: Additional file 2: Table S1. — Primer Sequences for bisulphite conversion efficiency experiments. Table S2. Bisulphite-pyrosequencing primer design for LDLR and SREBF1. Table S3. Methylation values per CpG within the SREBF1 amplicon measured by bisulphite-pyrosequencing. CpG 1 (in bold) denotes the cg2747935 Illumina 450k probe showing genome-wide significance between experimental groups. Table S4. Methylation values per CpG within the LDLR amplicon measured by bisulphite-pyrosequencing. CpG 2 (in bold) denotes the cg22971501 Illumina 450k probe showing genome-wide significance between experimental groups. No data was generated from CpG 4 due to poor sequencing efficiency. Table S5. Beta (methylation)-values at all Illumina 450k probes per experimental group located within the LDLR, SREBF1 and SREBF2 genes. The two probes where between-group methylation difference reaches genome-wide significance (FDR-adjusted P value < 0.05) are highlighted in yellow. [file 13148_2015_46_MOESM2_ESM.docx]

**Supplementary Methods**

**Bisulphite conversion efficiency:** The qPCR reactions were designed against parts of the MLH and GAPDH genes (see Supplementary Table 1 for primer design). The MLH primer pair is designed against the expected sequence of a region of the gene after the genetic changes brought about by successful bisulphite conversion. The GAPDH primer pair is designed to normal genomic sequence. Thus, if conversion is successful a product from the MLH1 primer pair should be seen and if it is not successful the template will not match the primer sequence and no product can result. Conversely the GAPDH primers should only produce product when conversion is NOT successful as this is when the primer sequences will properly match the template. The ratio of each of these products in a given sample can then be used to determine the conversion efficiency.

**Supplementary Table 1.** Primer Sequences for bisulphite conversion efficiency experiments

| Gene | **Target** | **Primer** | **Primer sequence** |
| --- | --- | --- | --- |
| MLH1 | Bisulphite converted sequence | F (5'>3') | GGAGTGAAGGAGGTTACGGGTAAGT |
|  |  | R (5'>3') | AAAAACGATAAAACCCTATACCTAATCTATC |
| GAPDH | Unconverted sequence | F (5'>3') | CGCCCCCGGTTTCTATAAAT |
|  |  | R (5'>3') | CAAAAGAAGATGCGGCTGAC |

**Supplementary Table 2**. Bisulphite-pyrosequencing primer design for LDLR and SREBF1

| Gene | **Primer** | **Primer sequence** | **Product Size (bp)** | **Chr-location** |
| --- | --- | --- | --- | --- |
| LDLR | Forward | TGATTTTGTTTTGAGGGAGAGGA | 192 | Chr19: 11,199,399-11,199,590 |
|  | Reverse | [Btn]ATAAAATCTTCACAAAAATACAACCAAA |  |  |
| SREBF1 | Forward | AGAGGTTTGTGTAGGTTTTAGAA | 156 | Chr17: 17,723,166  17,723,321 |
|  | Reverse | [Btn]CCCTACCTAAACTCTAATACAAATAC |  |  |

**Supplementary Results**

**Supplementary Table 3.** Methylation values per CpG within the SREBF1 amplicon measured by bisulphite-pyrosequencing. CpG 1 (in bold) denotes the cg2747935 Illumina 450k probe showing genome-wide significance between experimental groups.

| **SREBF1** | | | | | |
| --- | --- | --- | --- | --- | --- |
|  | **CpG 1** | CpG 2 | CpG 3 | CpG 4 | **Mean** |
| Normal B12 | **15.35** | 31.69 | 56.58 | 20.91 | **31.13** |
| Low B12 | **13.04** | 26.30 | 49.19 | 16.28 | **26.20** |
| No B12 | **12.59** | 27.72 | 49.38 | 17.14 | **26.71** |

**Supplementary Table 4.** Methylation values per CpG within the LDLR amplicon measured by bisulphite-pyrosequencing. CpG 2 (in bold) denotes the cg22971501 Illumina 450k probe showing genome-wide significance between experimental groups. No data was generated from CpG 4 due to poor sequencing efficiency.

| **LDLR** | | | | | |
| --- | --- | --- | --- | --- | --- |
|  | CpG 1 | **CpG 2** | CpG 3 | CpG 4 | **Mean** |
| Normal B12 | 83.12 | **42.22** | 29.24 | No data | **51.53** |
| Low B12 | 77.43 | **38.48** | 27.88 | No data | **47.93** |
| No B12 | 73.75 | **38.73** | 27.47 | No data | **46.65** |

|  | **Supplementary Table 5.** Beta(methylation)-values at all Illumina 450k probes per experimental group located within the LDLR, SREBF1 and SREBF2 genes. The two probes where between-group methylation difference reaches genome-wide significance (FDR-adjusted p-value <0.05) are highlighted in yellow. | | | | | | | | | | | |
| --- | --- | --- | --- | --- | --- | --- | --- | --- | --- | --- | --- | --- |
|  |  |  |  |  |  |  |  |  | **Mean beta-values per experimental group** | | |  |
|  | **Name** | **CHR** | **MAPINFO** | **Strand** | **Probe_SNPs** | **Probe_SNPs_10** | **UCSC_RefGene_Group** | **Relation_to_UCSC_CpG_Island** | **Group A** | **Group B** | **Group C** | **Strand** |
| **LDLR** | cg10201616 | 19 | 11198556 | F |  |  | TSS1500 | N_Shelf | 0.9306 | 0.9255 | 0.9248 | F |
|  | cg22971501 | 19 | 11199476 | F | rs36218923 |  | TSS1500 | N_Shore | 0.6503 | 0.6103 | 0.6033 | F |
|  | cg07960944 | 19 | 11199851 | F |  |  | TSS1500 | N_Shore | 0.0487 | 0.0457 | 0.0496 | F |
|  | cg05249393 | 19 | 11199883 | F |  |  | TSS200 | N_Shore | 0.1564 | 0.1572 | 0.1627 | F |
|  | cg22381454 | 19 | 11199903 | F |  |  | TSS200 | N_Shore | 0.0879 | 0.0879 | 0.0834 | F |
|  | cg18596381 | 19 | 11199917 | F | rs17249134 |  | TSS200 | N_Shore | 0.0924 | 0.0917 | 0.0945 | F |
|  | cg19751789 | 19 | 11199944 | R |  |  | TSS200 | N_Shore | 0.0996 | 0.1163 | 0.1044 | R |
|  | cg12013591 | 19 | 11199965 | F | rs17249141 |  | TSS200 | N_Shore | 0.0959 | 0.0909 | 0.0959 | F |
|  | cg23943360 | 19 | 11200146 | R |  |  | 1stExon;5'UTR | N_Shore | 0.0739 | 0.0767 | 0.0736 | R |
|  | cg13351962 | 19 | 11200983 | F |  |  | Body | Island | 0.0449 | 0.0433 | 0.0419 | F |
|  | cg06829293 | 19 | 11201108 | F | rs57217136 |  | Body | Island | 0.0518 | 0.0479 | 0.0507 | F |
|  | cg14331002 | 19 | 11201349 | F |  |  | Body | Island | 0.0268 | 0.0277 | 0.0284 | F |
|  | cg08826460 | 19 | 11201433 | F |  |  | Body | Island | 0.1891 | 0.1864 | 0.1850 | F |
|  | cg00919002 | 19 | 11201619 | F |  |  | Body | Island | 0.0995 | 0.0961 | 0.0900 | F |
|  | cg07512814 | 19 | 11203661 | R |  |  | Body | S_Shore | 0.7148 | 0.7033 | 0.6894 | R |
|  | cg13743731 | 19 | 11204447 | F |  | rs77238163 | Body | S_Shelf | 0.8710 | 0.8701 | 0.8756 | F |
|  | cg06950392 | 19 | 11213564 | R |  |  | Body | N_Shelf | 0.9416 | 0.9462 | 0.9451 | R |
|  | cg03554660 | 19 | 11214666 | R |  |  | Body | N_Shore | 0.8485 | 0.8422 | 0.8466 | R |
|  | cg21743867 | 19 | 11215999 | F |  |  | Body | Island | 0.8974 | 0.8971 | 0.8940 | F |
|  | cg24113439 | 19 | 11216129 | F |  |  | Body | Island | 0.9140 | 0.9136 | 0.9119 | F |
|  | cg27477601 | 19 | 11216203 | F |  |  | Body | Island | 0.8810 | 0.8705 | 0.8801 | F |
|  | cg17992670 | 19 | 11216306 | F |  |  | Body | Island | 0.7244 | 0.7291 | 0.7328 | F |
|  | cg10640764 | 19 | 11216364 | R |  |  | Body | S_Shore | 0.9290 | 0.9292 | 0.9225 | R |
|  | cg26313301 | 19 | 11219615 | R |  |  | Body | S_Shelf | 0.9352 | 0.9351 | 0.9339 | R |
|  | cg03567652 | 19 | 11242272 | R |  |  | 3'UTR |  | 0.3315 | 0.3314 | 0.3374 | R |
|  |  |  |  |  |  |  |  |  |  |  |  |  |
|  |  |  |  |  |  |  |  |  | **Mean beta-values per experimental group** | | |  |
|  | **Name** | **CHR** | **MAPINFO** | **Strand** | **Probe_SNPs** | **Probe_SNPs_10** | **UCSC_RefGene_Group** | **Relation_to_UCSC_CpG_Island** | **Group A** | **Group B** | **Group C** | **Strand** |
| **SREBF1** | cg23875758 | 17 | 17715520 | R |  |  | 3'UTR | N_Shore | 0.9312 | 0.9303 | 0.9334 | R |
|  | cg15996882 | 17 | 17715815 | F |  |  | 3'UTR | Island | 0.3123 | 0.3276 | 0.3196 | F |
|  | cg13721589 | 17 | 17716006 | F | rs1062463 |  | Body | Island | 0.9786 | 0.9795 | 0.9791 | F |
|  | cg03641529 | 17 | 17716218 | F |  |  | Body | Island | 0.5181 | 0.5130 | 0.5150 | F |
|  | cg11637976 | 17 | 17716852 | R |  |  | Body | S_Shore | 0.9018 | 0.9047 | 0.9085 | R |
|  | cg15863539 | 17 | 17716950 | R |  |  | Body | S_Shore | 0.8882 | 0.8810 | 0.8828 | R |
|  | cg13639244 | 17 | 17717006 | R |  |  | Body | S_Shore | 0.8861 | 0.8872 | 0.8884 | R |
|  | cg20544516 | 17 | 17717183 | F |  |  | Body | S_Shore | 0.6188 | 0.6218 | 0.6198 | F |
|  | cg24161106 | 17 | 17717208 | F |  |  | Body | S_Shore | 0.9874 | 0.9867 | 0.9870 | F |
|  | cg11393407 | 17 | 17717239 | F |  |  | Body | S_Shore | 0.9585 | 0.9558 | 0.9618 | F |
|  | cg09494646 | 17 | 17717252 | F |  |  | TSS200;Body | S_Shore | 0.9736 | 0.9713 | 0.9730 | F |
|  | cg13245539 | 17 | 17717265 | F |  |  | TSS200;Body | S_Shore | 0.9595 | 0.9618 | 0.9622 | F |
|  | cg07415388 | 17 | 17717276 | F |  |  | TSS200;Body | S_Shore | 0.7095 | 0.7168 | 0.7119 | F |
|  | cg03164243 | 17 | 17717894 | F |  |  | TSS1500;Body | S_Shore | 0.8725 | 0.8686 | 0.8732 | F |
|  | cg04805065 | 17 | 17717947 | F | rs56088670 | rs36027058 | TSS1500;Body | S_Shore | 0.9490 | 0.9459 | 0.9483 | F |
|  | cg09186408 | 17 | 17717967 | F | rs56088670 |  | TSS1500;Body | S_Shore | 0.9746 | 0.9740 | 0.9722 | F |
|  | cg19619576 | 17 | 17718525 | F |  |  | TSS1500;Body | S_Shelf | 0.6987 | 0.6930 | 0.7038 | F |
|  | cg16912637 | 17 | 17720273 | F | rs36215896 |  | Body |  | 0.9525 | 0.9515 | 0.9522 | F |
|  | cg09796270 | 17 | 17721594 | R |  |  | Body |  | 0.9323 | 0.9345 | 0.9358 | R |
|  | cg27407935 | 17 | 17723235 | F |  |  | Body | N_Shelf | 0.4655 | 0.4276 | 0.4041 | F |
|  | cg25999891 | 17 | 17724687 | R |  |  | Body | N_Shelf | 0.8804 | 0.8797 | 0.8809 | R |
|  | cg10415767 | 17 | 17726759 | F |  |  | Body | N_Shore | 0.8112 | 0.8078 | 0.8007 | F |
|  | cg15030378 | 17 | 17726965 | R |  | rs12936927 | Body | Island | 0.0225 | 0.0210 | 0.0212 | R |
|  | cg07097265 | 17 | 17727093 | F |  |  | Body | Island | 0.1052 | 0.1009 | 0.1017 | F |
|  | cg08323893 | 17 | 17727208 | R |  |  | Body | Island | 0.0322 | 0.0331 | 0.0297 | R |
|  | cg05666074 | 17 | 17727281 | F | rs12945414 | rs62064118 | Body | Island | 0.1299 | 0.1302 | 0.1250 | F |
|  | cg08129017 | 17 | 17728660 | F |  |  | Body | S_Shore | 0.5617 | 0.5757 | 0.5686 | F |
|  | cg11024682 | 17 | 17730094 | R |  | rs2350978 | Body | S_Shelf | 0.9150 | 0.9126 | 0.9164 | R |
|  | cg16657453 | 17 | 17735704 | F |  |  | Body | N_Shelf | 0.9184 | 0.9214 | 0.9185 | F |
|  | cg03963689 | 17 | 17739407 | F | rs78711184 |  | Body | N_Shore | 0.0655 | 0.0697 | 0.0717 | F |
|  | cg21099776 | 17 | 17739742 | R |  |  | Body | Island | 0.0662 | 0.0637 | 0.0702 | R |
|  | cg03628186 | 17 | 17739914 | R |  |  | Body | Island | 0.1811 | 0.1869 | 0.1779 | R |
|  | cg23159296 | 17 | 17740009 | F |  |  | Body | Island | 0.0216 | 0.0252 | 0.0236 | F |
|  | cg06261007 | 17 | 17740275 | F |  | rs13306736 | 1stExon;5'UTR | Island | 0.0245 | 0.0239 | 0.0272 | F |
|  | cg05810949 | 17 | 17740344 | F |  |  | TSS200 | Island | 0.0884 | 0.0968 | 0.0850 | F |
|  | cg21652344 | 17 | 17740365 | R |  |  | TSS200 | Island | 0.0388 | 0.0396 | 0.0365 | R |
|  | cg12079271 | 17 | 17740387 | F |  |  | TSS200 | Island | 0.0496 | 0.0463 | 0.0463 | F |
|  | cg00341606 | 17 | 17740405 | F | rs55861077 |  | TSS200 | Island | 0.1163 | 0.1043 | 0.1124 | F |
|  | cg14442061 | 17 | 17740468 | F |  |  | TSS200 | Island | 0.0998 | 0.1108 | 0.1213 | F |
|  | cg14808739 | 17 | 17741098 | R |  |  | TSS1500 | S_Shore | 0.7659 | 0.7661 | 0.7667 | R |
|  | cg16928487 | 17 | 17741425 | R |  |  | TSS1500 | N_Shore | 0.1744 | 0.1688 | 0.1730 | R |
|  | cg26588076 | 17 | 17741631 | F | rs7503739 |  | TSS1500 | N_Shore | 0.4392 | 0.4211 | 0.4192 | F |
|  |  |  |  |  |  |  |  |  |  |  |  |  |
|  |  |  |  |  |  |  |  |  |  |  |  |  |
|  |  |  |  |  |  |  |  |  | **Mean beta-values per experimental group** | | |  |
|  | **Name** | **CHR** | **MAPINFO** | **Strand** | **Probe_SNPs** | **Probe_SNPs_10** | **UCSC_RefGene_Group** | **Relation_to_UCSC_CpG_Island** | **Group A** | **Group B** | **Group C** | **Strand** |
| **SREBF2** | cg27535047 | 22 | 42228699 | R |  |  | TSS1500 | Island | 0.0457 | 0.0452 | 0.0420 | R |
|  | cg13637042 | 22 | 42228724 | R |  |  | TSS1500 | Island | 0.0434 | 0.0427 | 0.0400 | R |
|  | cg12856612 | 22 | 42228819 | R |  |  | TSS1500 | Island | 0.0491 | 0.0542 | 0.0514 | R |
|  | cg25937494 | 22 | 42228971 | F |  |  | TSS200 | Island | 0.0250 | 0.0244 | 0.0245 | F |
|  | cg20107653 | 22 | 42228989 | F |  |  | TSS200 | Island | 0.0566 | 0.0593 | 0.0572 | F |
|  | cg23067438 | 22 | 42228994 | F |  |  | TSS200 | Island | 0.0630 | 0.0645 | 0.0609 | F |
|  | cg27479400 | 22 | 42228998 | F |  |  | TSS200 | Island | 0.0561 | 0.0576 | 0.0548 | F |
|  | cg05509697 | 22 | 42229062 | R |  |  | TSS200 | Island | 0.0291 | 0.0312 | 0.0323 | R |
|  | cg25932461 | 22 | 42229068 | R |  |  | TSS200 | Island | 0.0244 | 0.0248 | 0.0247 | R |
|  | cg00830626 | 22 | 42229212 | R |  |  | 5'UTR;1stExon | Island | 0.0620 | 0.0607 | 0.0588 | R |
|  | cg07746918 | 22 | 42229324 | R |  |  | 1stExon | Island | 0.0612 | 0.0607 | 0.0621 | R |
|  | cg09978077 | 22 | 42229983 | F |  |  | Body | Island | 0.1131 | 0.1137 | 0.1108 | F |
|  | cg16000331 | 22 | 42230138 | R |  |  | Body | S_Shore | 0.1281 | 0.1270 | 0.1266 | R |
|  | cg21282452 | 22 | 42232160 | R |  |  | Body | S_Shelf | 0.9277 | 0.9323 | 0.9341 | R |
|  | cg21872942 | 22 | 42295667 | R |  |  | TSS1500;Body |  | 0.9364 | 0.9409 | 0.9383 | R |
|  | cg24260710 | 22 | 42296082 | F |  |  | TSS1500;Body |  | 0.9065 | 0.9065 | 0.9050 | F |
|  | cg26207035 | 22 | 42296553 | R |  |  | TSS1500;Body |  | 0.9279 | 0.9211 | 0.9188 | R |
|  | cg17473398 | 22 | 42296819 | F |  |  | TSS200;Body |  | 0.8853 | 0.8833 | 0.8890 | F |
|  | cg04181153 | 22 | 42296826 | F |  |  | TSS200;Body |  | 0.9110 | 0.9039 | 0.9103 | F |
|  | cg02233213 | 22 | 42296877 | F |  |  | TSS200;Body |  | 0.8724 | 0.8762 | 0.8730 | F |
|  | cg08401743 | 22 | 42296927 | F |  | rs59601893 | TSS200;Body |  | 0.9235 | 0.9262 | 0.9234 | F |
|  | cg12249359 | 22 | 42296941 | F |  |  | TSS200;Body |  | 0.8805 | 0.8865 | 0.8865 | F |
|  | cg16170490 | 22 | 42302146 | R |  |  | 3'UTR | N_Shelf | 0.9109 | 0.9098 | 0.9094 | R |
